# Pharmacologic Strategies for Post-Transplant Maintenance in Acute Myeloid Leukemia: It Is Time to Consider!

**DOI:** 10.3390/cancers14061490

**Published:** 2022-03-15

**Authors:** Iman Abou Dalle, Jean El Cheikh, Ali Bazarbachi

**Affiliations:** Hematology-Oncology Division, Department of Internal Medicine, American University of Beirut Medical Center, Beirut 1107 2020, Lebanon; ia41@aub.edu.lb (I.A.D.); je46@aub.edu.lb (J.E.C.)

**Keywords:** AML, maintenance, relapse, target, MRD

## Abstract

**Simple Summary:**

Allogeneic hematopoietic cell transplantation (allo-HCT) is a potentially curative therapeutic procedure for patients with high risk acute myeloid leukemia. Its anti-leukemic activity is mainly derived from the intensity of the conditioning regimen used and the graft-versus-leukemia effect exerted by the donor T lymphocytes. Despite major progress in the allo-HCT procedure in recent years with the achievement of lower non-relapse mortality and long-term disease control. However, up to 45% of patients still experience relapse. Therapeutic strategies post-transplant aiming at preventing disease relapse are discussed in this review.

**Abstract:**

Patients with high-risk acute myeloid leukemia are offered allogeneic hematopoietic cell transplantation (allo-HCT) in first remission to reduce risk of relapse. However, disease recurrence remains the major reason of allo-HCT failure, occurring in around 35–45% of patients, and leading to dismal outcomes. Strategies to reduce the risk of relapse are greatly needed, especially in the early post-transplant phase where the graft-versus-leukemia (GVL) effect is not yet activated. Some practices include the use of myeloablative conditioning regimens, close monitoring of measurable residual disease and donor chimerism, rapid tapering of immunosuppression, and implementation of pre-emptive strategies as the use of donor lymphocyte infusion. However, it’s time to consider prophylactic pharmacologic interventions post allo-HCT that aim at maintaining leukemic clones under control by both direct cytotoxic activity and by enhancing the GVL effect. In this current review, available data on drugs targeting epigenetic pathways like azacitidine, or actionable mutations like FLT3 and IDH1/2 inhibitors used as maintenance post allo-HCT, will be discussed.

## 1. Introduction

Allogeneic hematopoietic cell transplantation (allo-HCT) could serve as a curative treatment in a subset of patients with acute myeloid leukemia (AML). According to the European Leukemia Network (ELN) 2017 recommendations, patients with AML at high risk of relapse, exceeding 35–40% if continued on cytotoxic chemotherapy alone, should be offered allo-HCT, as the risk of non-relapse mortality (NRM) is allowable in order to mitigate the expected relapse risk [1]. The dynamic risk stratification of the patients takes into consideration cytogenetic and molecular data, as well as the status of measurable residual disease (MRD) after chemotherapy [1].

The ultimate goal for a successful allo-HCT in AML is a reduction in both NRM and relapse risk, translated into an improved overall survival (OS). In the past decade, many new advances in allo-HCT techniques expanded its use worldwide, including reduced-intensity conditioning regimens allowing allo-HCT in patients up to 75 years old, growing donor availability through international donor registries and haplo-identical donors, better graft-versus-host disease (GVHD) prophylaxis, especially in vivo T cell depletion and post-transplant cyclophosphamide, and improved supportive care strategies. Jointly, these developments ensure a potentially less toxic allo-HCT procedure. However, despite improvements in NRM and supportive care, patients with AML are still at high risk of relapse post allo-HCT with cumulative incidence of relapse at 2 years ranging between 30 and 70% depending on the type of conditioning regimen and the pre-transplant disease status [2,3,4]. Those patients who relapse post allo-HCT have generally poor prognosis, especially in the early post-transplant period. In the recent years, the two-year OS of patients relapsing post allo-HCT was steadily improving from 16% to 26% among young adults, but there is still a long way to go [5].

Allo-HCT is a procedure that relies on both the anti-leukemic effect of the conditioning regimen and the allo-immunity generated by the graft against the leukemia cells. To further reduce relapse risk, post-transplant interventions that possess a direct cytotoxic activity and are capable of simultaneously regulating the immune environment toward the graft-versus-leukemia (GVL) direction are much needed. Concomitantly, these strategies should take into consideration the delicate state post allo-HCT in terms of cytopenias, increased risk of infections, risk of graft-versus-host disease (GVHD) and drug-drug interactions.

## 2. Prophylaxis or Pre-Emptive?

Several strategies have been adopted to prolong disease-free survival after allo-HCT. Based on the time of intervention, they can be categorized into either preemptive approaches—those commenced at the time of detection of MRD—or prophylactic approaches—those initiated in the absence of detectable leukemia. Accumulated data clearly suggest that pre- and post-transplant detectable MRD, either by multiparametric flow cytometry (MFC) or potentially by next-generation sequencing (NGS), are strongly associated with an increased cumulative incidence of relapse (CIR) [6,7,8]. The FIGARO study demonstrated a significantly increased two-year CIR in patients with pre-transplant detectable MRD by both conventional and computational MFC assays after reduced-intensity allograft (41% versus 20%, *p* = 0.01) when compared to MRD-negative patients [9]. In another study using NGS-based MRD assays, patients with positive MRD post-transplant had a significantly higher five-year CIR compared to MRD-negative patients (53% versus 26%, *p* < 0.001) [8]. Subsequently, pre-emptive treatment could be considered for those patients with imminent relapse. Donor-recipient chimerism can also be followed in the post-transplant setting, and used as a tool to predict disease relapse, as dropping donor chimerism (particularly below 95%) can precede MRD positivity and is also associated with increased risk of relapse [10,11,12]. Moreover, early acquisition of full chimerism could overcome the negative effect of pre-transplant MRD positivity and improve outcomes regardless of the conditioning regimen used as supported by the FIGARO study [9]. Pre-emptive use of donor lymphocyte infusion (DLI) in patients with mixed chimerism may improve their outcomes with a nevertheless higher risk of GVHD depending on the dose and numbers of DLI.

The use of conventional MFC assays by assessing aberrant leukemia-associated immunophenotypes (LAIPs) or by the “different-from-normal” (DfN) approach to determine the presence of MRD in AML has long been debated for subjectivity and lack of standardization. Many attempts are being made to improve on MRD sensitivity in AML for better outcome prediction [13]. The leukemia stem cell (LSC) based assays were investigated in the post-transplant setting using a combination of CD7, CD11b, CD22, CD56, Tim-3, and CLL-1 on CD34 + CD38- cells including 360 patients [14]. Patients with post-transplant detectable MRD by LSC-based assay had a higher CIR of 42.7% versus 2.6% for LSC MRD-negative patients (*p* < 0.001). Compared with MRD detected by the conventional MFC assay, using LSCs for MRD evaluation had also a higher sensitivity (66.7% versus 43%) [14].

The European LeukemiaNet (ELN) MRD Working Party has recently published the 2021 update on MRD evaluation in AML, as a prognostic and predictive tool for risk assessment and treatment decision-making [15]. Their recommendations for MRD evaluation integrated the use of NGS-MRD assays for unique molecular identifiers and explored the potential benefit of LSC MFC assays to be further validated in clinical trials [15].

Post-transplant prophylactic treatment is used to complement the effect of allo-HCT, either by augmenting the direct anti-leukemia activity, or by enhancing the GVL effect. The choice of the drug in this setting should take many aspects into consideration. First, the drug should be effective with known anti-leukemia activity and the capability of eradicating any emerging resistant leukemia clones. Second, it should have an acceptable safety profile with no significant increase in risk of cytopenias, infections, GVHD, and NRM. Third, it is preferable to be convenient for patients, as with oral formulations, for example, that can limit hospital visits and improve quality of life.

## 3. Patient 1: Secondary AML with Adverse Cytogenetics

A 63-year-old man patient known to have diabetes mellitus type II was diagnosed in August 2012 with secondary AML arising from myelodysplastic syndrome and complex cytogenetics. He received four cycles of azacitidine with a repeated bone marrow biopsy showing dysplasia and persistent 8% blasts. The patient underwent matched related allo-HCT with myeloablative conditioning with fludarabine, busulfan and thymoglobin in January 2013. His evaluation at day 30 post allo-HCT showed cellular marrow with trilineage hematopoiesis, normal cytogenetics and full donor chimerism. The patient had no signs of GVHD post allo-HCT. He was started on azacitidine maintenance 32 mg/m^2^ for five days every 28 days starting at day + 45. The patient continued five years of azacitidine with continuous complete remission, then opted to stop maintenance in May 2018. Six months later, the patient presented with relapsed AML with myelodysplastic related changes and 70% blasts. He was re-induced with cytotoxic chemotherapy including high dose cytarabine and cladribine, achieved remission, followed by a second allo-HCT, but unfortunately died a few weeks later due to transplant complications.

## 4. Maintenance with Hypomethylating Agents

Both azacitidine and decitabine are epigenetic modulators among the non-targeted agents that have been used in the post-transplant setting. It is believed that besides their anti-leukemia activity, hypomethylating agents can expand regulatory T cells by inducing FOXP3 expression on CD4 + CD25- T cells and thus reduce the risk of GVHD while enhancing the GVL effect [16,17,18].

Azacitidine has been an effective hypomethylating agent re-inducing remissions in post allo-HCT overt relapse in 25% of treated patients, with a two-year OS of 29% in responders [19]. The time to relapse and the number of blasts in the bone marrow were two independent predictive factors of survival [19]. Considering the low rate of response of azacitidine alone in the setting of overt relapse post allo-HCT because of a higher bulk of disease, pre-emptive treatment with azacitidine might produce better clinical responses in a larger subset of patients. Both RELAZA and RELAZA−2 trials proved that azacitidine at full doses (75 mg/m^2^ for 7 days) given pre-emptively in AML patients post allo-HCT based on their MRD status followed either by polymerase chain reaction (PCR) or donor chimerism was successful in preventing or delaying relapse [20,21]. With a median follow-up of 13 months after the start of MRD-guided treatment, relapse-free survival (RFS) at 12 months was 46% (95% confidence interval (CI) 32–59%) in the 53 patients who were MRD-positive and received azacitidine [21]. In patients with negative MRD who were not treated with azacitidine, the 12-month RFS was 88% (95% CI: 82–94%, *p* < 0.0001). These results suggest that azacitidine might work better in a smaller bulk of disease, so was then evaluated as a prophylaxis strategy.

In a dose-finding study, 45 patients with high-risk AML were treated with a five-day azacitidine regimen at different escalated doses (8, 16, 24, 32 and 40 mg/m^2^) for a maximum of four cycles. The use of low dose azacitidine at 32 mg/m^2^ for five days was found to be the optimal dosing, and correlated with a one-year OS of 77% in heavily pre-treated patients [22]. In another phase I/II trial, 27 AML patients post-reduced intensity conditioning (RIC) allo-HCT received azacitidine 32 mg/m^2^ for five days every 28 days for up to 10 cycles. Azacitidine treatment was very well tolerated except for mild hematologic adverse events, and was associated with lower incidence of GVHD [18]. In the RICAZA trial, 37 of 51 (73%) AML patients post allo-HCT received azacitidine maintenance after a median time of 54 days; of them 16 (43%) relapsed after a median time of eight months after allo-HCT. Interestingly, patients who had a CD8+ T-cell response following azacitidine treatment had a lower relapse rate, consistent with the hypothesis that azacitidine is capable of augmenting the alloreactive response and therefore the GVL effect [23]. Our group reported on 18 patients with high risk myeloid malignancies who received low dose azacitidine maintenance post allo-HCT. The median number of azacitidine cycles was 16 (range: 1–45 cycles), and none of the patients discontinued the drug because of toxicity. The one-year disease-free survival and OS were 63% and 70%, respectively [24].

These promising results needed a confirmatory phase III randomized trial that was recently published by Oran et al. [25]. This trial was slow to recruit, as it took nine years to recruit a total of 187 patients randomized to receive azacitidine at 32 mg/m^2^ for five days every 28 days for up to 12 cycles or no intervention. The median duration of treatment was short (4 months), with 60% of patients discontinuing treatment due to relapse or toxicity. The maintenance treatment failed to show any improvement in both RFS and OS (hazard ratios (HR) of 0.73 (95% CI, 0.49–1.1; *p* = 0.14 and 0.84 (95% CI, 0.55–1.29; *p* = 0.43) respectively [25].

Decitabine was also investigated in a dose escalating trial as post-transplant maintenance at doses ranging between 5 and 15 mg/m^2^ for five days every six weeks for a total of eight cycles [26]. Twenty-four patients were treated, and 75% of them experienced grade three and four hematologic toxicities. The two-year OS was encouraging at 56%, with a two-year CIR of 28% [26]. A recent Chinese open-label randomized trial investigated the use of recombinant human granulocyte colony stimulating factor (rh-GCSF) combined with low dose decitabine (5 mg/m^2^ for 5 days) versus no intervention in 204 patients with high-risk AML in the post allo-HCT setting [27]. The rationale of combining an agent that promotes cell cycle entry like rh-GCSF could augment the anti-leukemic effect of decitabine, in addition to the possibility of enhancing the function of cytotoxic CD8+ T cells, and T reg cells, thus improving the GvL effect [27]. After a median follow-up of 28 months, there was a significant reduction in estimated two-year CIR with the rh-GCSF and decitabine arm compared to the control arm (15% versus 38%, *p* < 0.01), which translated into a significant improvement in both two-year leukemia-free survival (LFS) and OS (82% versus 61%, *p* < 0.01 and 86% versus 70%, *p* = 0.01, respectively) [27].

The oral formulation of azacitidine (CC−486) may enhance patient convenience, eliminate injection-site reactions, and facilitate long-term administration. The application of this product in a post-allo-HCT setting has since been verified in a phase I/II study, which supports the promising clinical activity. The best effective and tolerable regimen was determined to be 200 mg orally daily for 14 days with 52% of patients having completed the planned 12 month cycles. The observed one-year CIR was 13%, with a median OS that was not reached [28]. A randomized, phase III trial to validate its efficacy is in development.

A recent systematic review explored the safety and efficacy of maintenance treatment following allo-SCT in AML and MDS [29]. It demonstrated rates of 65.6% and 56.2% for the two-year OS and RFS, respectively, of hypomethylating agents-treated patients. In addition, acute and chronic GVHD were found in 39.9% and 44.4% of patients, respectively [29].

**Figure 1:**
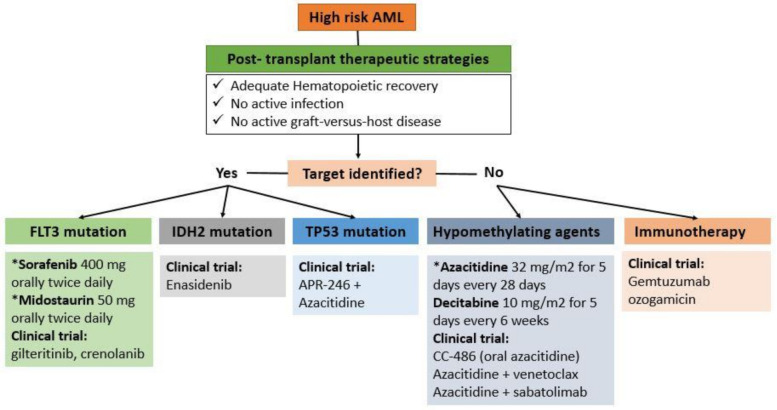
Therapeutic options for post-transplant maintenance in high risk AML. * recommended in clinical practice based on published clinical data.

Finally, the advantage of hypomethylating agents is that they could be widely used across all subtypes of AML regardless of genetic mutations, and they are currently used as the backbone for many potential combinations, either with DLI or targeted agents. (Figure 1).

## 5. Patient 2: A Young Patient with FLT3-ITD Mutant AML in First Remission 

A 43-year-old male patient diagnosed in April 2019 with AML presented with a white blood count of 129 × 10^9^/L with 62% circulating blasts. Genomic analysis revealed normal cytogenetics, with *NPM1* wild type, and detectable *FLT3*-ITD. He received 7 + 3 induction (daunorubicin 60 mg/m^2^ for days one to three and cytarabine 200 mg/m^2^ per day on days one to seven) in addition to midostaurin 50 mg twice daily on days eight to 21 of induction in May 2019. He achieved complete remission with full count recovery and continued with consolidation using high dose cytarabine 3 g/m^2^ twice daily on days one, three, and five, combined with midostaurin 50 mg twice daily on days eight to 21. He underwent a brother HLA full matched allo-HCT in August 2019 using a myeloablative conditioning. Evaluation at day +30 post allo-HCT revealed complete molecular remission with 98% donor chimerism. Patient had no signs of acute GVHD, and has been on sorafenib 400 mg orally twice daily for almost three years now. The patient continues to be in complete remission with no adverse events.

## 6. Maintenance with FLT3-Inhibitors

Activating mutations in the fms-like tyrosine kinase 3 (*FLT3*) occur in 30% of newly diagnosed AML patients, considered to be one of the most recurring mutations in AML. Patients usually present with a higher white cell count suggestive of a highly proliferating disease. *FLT3*-mutant AML is also associated with higher risk of relapse and worse survival compared to the *FLT3* wild type AML [30]. A consensus was made to offer consolidation with allo-HCT for all patients with *FLT3*-mutant AML [1]. Patients with lower allelic burden in the presence of the *NPM1* mutation have better outcomes when treated with intensive chemotherapy in addition to a FLT3 inhibitor, and may be spared an allo-HCT, although this recommendation is still controversial [31]. Among patients with *FLT3*-mutant AML who receive allo-HCT, the relapse incidence at two years is 30% to 50% [32]. Thus, preventing disease relapse in this subcategory of patients was considered an unmet need, where many studies incorporating post allo-HCT strategies are being evaluated.

In general, the outcomes of patients with *FLT3*-mutant AML have improved over time, especially with the introduction of FLT3 inhibitors. The RATIFY trial included newly diagnosed patients with *FLT3*-mutant AML and were randomized to receive intensive chemotherapy in addition to either midostaurin or placebo [33]. Those who received midostaurin as part of their induction and consolidation cycles had 22% reduction in their risk of death and a higher chance of proceeding to allo-HCT. However, midostaurin maintenance post allo-HCT was not allowed in the RATIFY trial [33].

Different studies incorporated FLT3 inhibitors as maintenance treatment post allo-HCT in *FLT3*-mutant AML. Sorafenib is a multi-targeted tyrosine kinase that was used and approved in renal cell carcinoma and hepatocellular carcinoma. It was long used as off-label in the treatment of AML, and the first FLT3 inhibitor to be tested in post allo-HCT setting in multiple case series, then investigated in phase I trial, retrospective and registry-based studies [34,35,36,37].

Besides its known anti-leukemia efficacy through FLT3 inhibition, sorafenib was shown to synergize with the allo-immune environment promoting a GVL effect through IL−15 production in mice and humans [38,39]. In the phase I dose-finding trial, 22 patients were treated with sorafenib at escalated doses until the maximal tolerated dose of 400 mg twice daily. Sorafenib was given safely after allo-HCT with a one-year PFS and OS of 85% and 95% deserving further investigation [40].

Multiple retrospective studies have followed this phase I trial, confirming the safety and efficacy of sorafenib maintenance post allo-HCT [35,37,41]. An EBMT registry-based analysis included 462 *FLT3*-mutant AML who had allo-HCT in first remission; 28 of these patients received maintenance sorafenib for a median duration exceeding 12 months. A matched-pair analysis demonstrated a significantly improved two-year OS (83% versus 62%) and two-year PFS (79% versus 54%) when compared with patients who did not receive any maintenance treatment [42].

In a phase II randomized trial (SORMAIN), 83 patients were randomly assigned to either sorafenib maintenance or placebo starting 60 to 100 days post-transplant for 24 months duration or until disease progression or intolerability. There was a 61% reduction in risk of relapse or death from sorafenib maintenance when compared to placebo (HR = 0.39, 95% CI: 0.18–0.85, *p* = 0.013). After a median follow-up of 42 months, the 2-year RFS was 85% in the sorafenib group versus 53.3% in the placebo group [43]. Although a minority of patients were treated with FLT3 inhibitor during the pre-transplant induction-consolidation phase, none of the patients with pre-transplant MRD negative disease relapsed while receiving sorafenib maintenance. This is indicative that sorafenib maintenance could still play a role even in the modern era where all *FLT3*-mutant AML patients are receiving upfront standard chemotherapy with the FLT3 inhibitor. Patients who were treated with sorafenib had a higher rate of acute and chronic GVHD (76.8% in sorafenib group versus 59.8% in the control group), which could also explain the effect of sorafenib on allo-immunity. Other toxicities included increased infections, GI and skin toxicities. 

A larger phase III randomized trial included 202 *FLT3*-mutant AML investigating sorafenib maintenance post allo-HCT; 100 patients were treated in the sorafenib arm versus 102 in the control arm. Sorafenib was given at day 30–60 post allo-HCT until day 180. Clearly, sorafenib maintenance resulted in an improved two-year OS (82% versus 68%) and two-year PFS (79% versus 56%) when compared to placebo [44]. Despite the non-approval of sorafenib in the treatment of AML, its effectiveness in the post-transplant setting was proven by multiple studies. In the absence of other approved FLT3 inhibitors in this setting, many authorities have recommended its off-label use [45,46].

Midostaurin is another multikinase inhibitor that targets both *FLT3*-ITD and *FLT3*-TKD mutations. It was approved by the FDA in April 2017 based on the CALGB 10603/ RATIFY trial to be given in addition to standard induction and consolidation treatment in adult patients aged less than 60 years old, as it showed significant improvement in OS [33]. Patients who were ineligible for allo-HCT could continue on single agent midostaurin 50 mg twice daily for an additional year as maintenance treatment; however, post allo-HCT maintenance with midostaurin was not allowed according to the trial protocol [33].

In the AML-SG 16–10 trial, 284 adult patients were treated in a phase II trial, 198 patients were 18 to 60 years old and 86 were older patients aged 61 to 70 years [47]. Treatment consisted of intensive chemotherapy in addition to midostaurin followed by allo-HCT followed by maintenance midostaurin for 12 months. 134 patients were able to receive allo-HCT, and 75 (56%) patients succeeded to continue on maintenance midostaurin after a median 71 days (range: 31–100 days). Maintenance was given for a median duration of nine months (range: 1–13 months), less than what was planned, mainly because of adverse events, including GI toxicity (80%), infections (56%), and cytopenias (52%). In a landmark analysis including patients who received allo-HCT and remained event-free in the first 100 days, those who received midostaurin maintenance had improved EFS and OS compared to those who did not (*p* = 0.004 and *p* = 0.01, respectively) [47]. Compared to historical controls from 5 AMLSG prospective trials, EFS was significantly higher in patients treated with midostaurin maintenance both in younger and older populations with a hazard ratio of 0.58 (95% CI, 0.48–0.70; *p* < 0.001) [47].

The RADIUS trial is another phase II randomized open label trial specifically evaluating the safety and efficacy of midostaurin in the post-transplant setting [48]. Although the study was not powered to detect a difference in survival, it was an early evidence that midostaurin maintenance could also work in this setting [48]. Sixty adult patients with *FLT3*-mutant AML after undergoing allo-HCT in first remission were randomized to receive either standard of care (n = 14) or midostaurin 50 mg twice daily for 12 months (n = 16). The median time on treatment was 10 months, and most adverse events were from GI toxicity. The 18-month RFS was 89% in the midostaurin arm and 76% in the control arm with a hazard ratio of 0.46 (95% CI, 0.12–1.86; *p* = 0.27). The rate of GVHD was similar between the two arms.

Both sorafenib and midostaurin are multikinase inhibitor including FLT3 and multiple other kinases. Second generation FLT3 inhibitors were developed with higher potency against FLT3 tyrosine kinase to be evaluated in both frontline and relapsed/refractory (R/R) *FLT3*-mutant AML. In the post-transplant setting, there is now a better understanding that the effectiveness of maintenance is not only dependent on FLT3 inhibition, but also on manipulating the GVL effect. In one study, phosphorylated FLT3 was used as a biomarker of clinical outcomes; patients who attained less than 70% phosphorylated FLT3 had better outcomes than others with higher phosphorylated FLT3, consistent with the hypothesis that FLT3 potency might play a role in reducing relapses post allo-HCT [48].

Quizartinib, a second generation potent FLT3-inhibitor, was evaluated in a phase I dose finding trial. Thirteen patients with *FLT3*-mutant AML in remission post allo-HCT were treated with two different doses (40 mg and 60 mg per day) every 28 days for a total of 24 months. The majority of patients received at least one year of treatment. One third of patients discontinued because of adverse events, and only one patient (8%) relapsed during the first cycle [49]. This study showed some evidence that potent FLT3 inhibitors are capable of preventing relapse, with an acceptable toxicity profile. Given the FDA rejection of quizartinib as single agent in the treatment of R/R *FLT3*-mutant AML, further prospective randomized trials in the post-transplant setting might not be undertaken in the near future. The other path for approval might be through combination regimens with chemotherapy or hypomethylating agents and venetoclax. However, another second-generation potent FLT3 inhibitor active against both *FLT3*-ITD and *FLT3*-TKD mutations, currently FDA approved in the R/R AML setting, is gilteritinib, based on the ADMIRAL trial [50].

One of the ongoing randomized trials is the Blood and Marrow Transplant Clinical Trials Network (BMT CTN) 1506 (NCT02997202) [51]. It is a multicentric Phase 3 randomized double blinded trial evaluating the efficacy of maintenance gilteritinib in the post-transplant phase randomized to placebo, which opened accrual in 2017. The target enrollment is 346 adult patients. Aside from its higher FLT3 potency, gilteritinib is thought to be better tolerated than other multikinase inhibitors. Pre-transplant MRD assessment by NGS based assays for *FLT3* mutation will be studied as a biomarker of response [51]. The trial is still ongoing and results are expected soon. While there is confidence that this trial will meet its primary endpoint and show superiority of gilteritinib over placebo as post-transplant maintenance, the debate will be ongoing over which FLT3 inhibitor to choose post allo-HCT as the control arm in this trial was placebo and not sorafenib [52]. The decision will definitely be based on drug availability, tolerability, MRD status pre-and post-transplant and *FLT3* mutation type.

The optimal duration of maintenance treatment in the post allo-HCT setting is not very well established and is considered another ongoing debate. Until now, it is unclear if maintenance treatment prevents disease relapse to allow enough time for a GVL response post allo-HCT, or it is just delaying it. It was evident from multiple maintenance studies that longer duration of treatment was associated with a better relapse-free state. Patients treated with azacitidine maintenance needed at least three cycles of treatment to show T-cell responses and therefore improvement in LFS [23]. Moreover, in the phase III randomized trial conducted by Oran et al., no improvement in both RFS and OS were observed when patients received a median four months of treatment [25]. The same observations were made when analyzing the RFS and OS events beyond two years of the SORMAIN trial, where sorafenib maintenance was given for 24 months duration. Those events might be preventable with longer duration of maintenance treatment [43]. In the EBMT consensus practice recommendation, maintenance treatment with sorafenib in *FLT3*-mutant AML was encouraged for all patients for at least two years, depending on tolerability [45] (Figure 1).

## 7. Maintenance with Promising Novel Agents 

Exciting times in the field of AML happened in the last five years; many targeted novel agents have been investigated and approved in the treatment of AML either in the frontline or in the R/R settings. Those novel agents are ideal candidates for maintenance treatment post allo-HCT, as these are orally available, and their continuous use has been proven to be safe and tolerable. 

In *IDH1/2* mutant AML, both ivosidenib and enasidenib are targeted agents inhibiting mutant IDH1 and IDH2, respectively [53,54]. They were approved in the R/R setting and are currently being investigated in combination with chemotherapy or hypomethylating agents in the frontline setting. Both drugs are being evaluated in phase I trials to determine their tolerability as maintenance therapy post allo-HCT (NCT03564821, NCT03728335).

Venetoclax, an orally available selective BCL2-inhibitor, is another breakthrough targeted agent that changed the landscape of AML treatment. It is currently used in the frontline treatment of elderly patients with AML in combination with low dose cytarabine or hypomethylating agents based on two pivotal trials, VIALE-A and C trials [55,56]. A small study that included 23 AML patients with detectable MRD at time of transplant reported preliminary results on single agent venetoclax maintenance given initially at 100 mg daily, then uptitrated to a maximum of 400 mg daily for up to one year. Although preliminary data suggested that venetoclax maintenance is tolerable in the post allo-HCT setting with no unexpected side effects, almost half of the patients had to interrupt or dose-reduce venetoclax due to adverse events, mainly cytopenias and diarrhea [57]. Two ongoing Phase 2 and 3 clinical trials (NCT04128501, NCT04161885) are currently recruiting patients to investigate the combination of azacitidine with venetoclax in the post-transplant setting (Table 1). One of the noted differences between the two trials is the duration of venetoclax treatment; while it is limited to seven days in the phase 2 trial, it will continue over 28 days in the randomized VIALE-T trial. Results will be eagerly awaited, especially concerning the risk of myelosuppression and graft function.

Although this review focuses on the pharmacologic strategies to reduce AML relapse post allo-HCT, we should mention the role of cellular therapies either alone or in combination with the above-mentioned pharmacologic interventions. Prophylactic DLI may be effective in patients with high-risk AML or in those transplanted beyond first remission [58]. Moreover, a repetitive low-dose of prophylactic DLI over several years will help in preventing relapses, with a reduced risk of DLI-mediated GVHD [59,60,61].

Targeting the leukemia surface antigens in AML either by antibody-drug conjugates, bispecific T-cell engagers, and CAR T cell therapies is gaining interest and is currently being evaluated in many preclinical and clinical trials, mainly in the relapsed/refractory setting [62]. Unlike B-cell acute lymphoblastic leukemia, where CD19 can be considered an ideal target with acceptable on-target off-tumor effect leading to B-cell aplasia, an optimal target in AML still represents a challenge. Many surface antigens overexpressed on AML cells and LSCs are being investigated and can potentially be targeted, such as CD33, CD123, and CLL1, Tim-3 [63].

## 8. Conclusions

While allo-HCT is potentially curative in patients with high-risk AML, it should not be regarded as the final stage in the treatment plan, as many patients still relapse. Multiple strategies can be implemented to reduce the risk of relapse, especially in the high-risk population, based on their cytogenetics, molecular risks, and their pre- or post-transplant MRD status. Pharmacologic interventions given early after engraftment will maintain their anti-leukemia effect, particularly after the use of a reduced-intensity conditioning in the meantime until a GVL effect is mounted. Both drugs targeting the epigenetic pathway, such as azacitidine, or actionable mutations like FLT3 inhibitors demonstrated efficacy and safety with the current available data. Their use is recommended in the absence of acute GVHD for at least two years, as long as these are tolerated. There is a lot of enthusiasm related to the emergence of multiple novel agents currently being incorporated in the management of AML and may be considered in the future for post-transplant maintenance. Taking into consideration many challenges that randomized trials could face in the post-transplant management setting, these remain the right path to solve many unanswered questions regarding which patient should be treated, what is the optimal agent to be used, and for how long maintenance is needed. Until we have clear answers, it’s probably time to consider maintenance intervention for all high-risk AML patients.

## Figures and Tables

**Table 1:** Selected ongoing trials evaluating post-transplant maintenance treatments in acute myeloid leukemia.

Treatment	Study Phase	Timing of Intervention	Study Status	Clinical Trial Identifier
**FLT3 Target**				
Gilteritinib	Phase 3	Prophylaxis	Active, not recruiting	NCT02997202
Crenolanib	Phase 2	Prophylaxis	Active, not recruiting	NCT02400255
**IDH2 Target**				
Enasidenib	Phase I Phase I	Prophylaxis	Recruiting Active, not recruiting	NCT03728335 NCT03515512
**TP53 Target**				
APR−246 + azacitidine	Phase 2	Prophylaxis	Active, not recruiting	NCT03931291
**CD 33 Target**				
Gemtuzumab ozogamicin	Phase 1/2	Prophylaxis	Recruiting	NCT04849910
**No Target Identified**				
Azacitidine + venetoclax	Phase 3 VIALE-T	Prophylaxis	Recruiting	NCT04161885
	Phase 2	Prophylaxis	Recruiting	NCT04128501
	Phase 2	Pre-emptive	Recruiting	NCT04809181
Azacitidine + Pevonedistat	Phase 2	Pre-emptive	Recruiting	NCT04712942
Azacitidine + Valproic acid	Phase 2	Prophylaxis	Recruiting	NCT02124174
SGI−110 + DLI	Phase 2	Prophylaxis	Not yet recruiting	NCT03454984
Low dose 5-azacitidine	Phase 2	Prophylaxis	Recruiting	NCT01995578
Oral Azacitidine (CC−486)	Phase 3	Prophylaxis	Recruiting	NCT04173533
Sabatolimab ± Azacitidine	Phase Ib/II	Pre-emptive	Recruiting	NCT04623216
Glasdegib	Phase 3	Prophylaxis	Recruiting	NCT04168502
N−803 IL−15 super-agonist complex	Phase 2	Prophylaxis	Recruiting	NCT02989844
